# Scientific impact increases when researchers publish in open access and international collaboration: A bibliometric analysis on poverty-related disease papers

**DOI:** 10.1371/journal.pone.0203156

**Published:** 2018-09-19

**Authors:** J. Gabrielle Breugelmans, Guillaume Roberge, Chantale Tippett, Matt Durning, David Brooke Struck, Michael M. Makanga

**Affiliations:** 1 European & Developing Countries Clinical Trials Partnership (EDCTP), The Hague, Netherlands; 2 Science-Metrix, Montreal, Quebec, Canada; Institut Català de Paleoecologia Humana i Evolució Social (IPHES), SPAIN

## Abstract

**Background:**

The European & Developing Countries Clinical Trials Partnership (EDCTP), like many other research funders, requires its grantees to make papers available via open access (OA). This article investigates the effect of publishing in OA journals and international collaboration within and between European and sub-Saharan African countries on citation impact and likelihood of falling into the top 1% and top 10% most cited papers in poverty-related disease (PRD) research.

**Methods:**

Disease-specific research publications were identified in the Web of Science™ and MEDLINE using Medical Subject Heading (MeSH) terms. Data on the open accessibility of scientific literature were derived from 1science oaFindr. Publication data, including relative citation counts, were extracted for 2003–2015. Regression models were applied to quantify the relationship between relative citations and presence in the 1% and top 10% most cited papers versus OA and international collaboration.

**Results:**

The results show that since 2003 papers on PRDs have become increasingly available in OA. Among all PRD areas, malaria research is most frequently published in OA and in international collaboration. The adjusted regression analyses show that holding other factors constant, publishing research in OA and in international collaboration has a significant and meaningful citation advantage over non-OA or non-international collaborative research. Publishing papers as part of a European-wide or European- sub-Saharan African collaboration increases research impact. In contrast, such collaboration advantage is not observed for research output involving sub-Saharan Africa only which seems to decrease research impact.

**Conclusions:**

Our results indicate that there is a real, measurable citation advantage for publishing PRD research in OA and international collaboration. However, the international collaboration advantage seems to be region-specific with increased research impact for European-wide and European-sub-Saharan African collaborations but a decrease in research impact of collaborations confined to sub-Saharan African research institutions. Further research is required to further verify this finding and to understand the underlying factors related to this observed decrease in research impact. To target future research capacity building activities in sub-Saharan Africa it is important to assess whether the observed decreased impact reflects the scientific competencies and geographic distribution of individual researchers or institutional-, national- or funder-specific research requirements.

## Introduction

With the advent of the Internet, scientific publishing has changed profoundly over the last twenty years. Where it was typical for many scientists in the nineties to be subscribed to various scientific journals and to own physical volumes, today almost all scientific papers are offered and retrieved in digital format. When paper issues were the only available option, a wide enough subscriber base was a condition for sustainability of a journal [1]. However, with the expansion of Internet-enabled innovations such as low-cost distribution of scientific content, some scholars and publishers have opted for new business models where anybody with Internet access can have free, immediate and unrestricted online access to peer-reviewed scientific literature. This model is known as the “open access” (OA) model. Income models used to support the OA journals differ from those of the traditional subscription-based journals in that the required resources to operate are collected by means other than charging the reader [2]. For example, many OA journals charge authors for submissions or rely on advertising revenue as a source of income.

There are several ways to make research available in OA. Two frequently cited models of OA are "green" and "gold" [3]. Green (self-archiving) is when an author before, simultaneously or after appearance in the journal, self-archives a version of the article for free public use in their institutional repository, in a central repository (e.g., PubMed Central), or on some other website [3]. For gold OA (publishing in an OA Journal), the article is published in an OA journal that provides immediate OA to all its papers on the publisher's website [3]. The gold model uses a traditional journal publication system, but shifts the economic/financial model [4]. Instead of a subscriber paying to read the final version of a peer-reviewed article, an author or sponsor pays to publish the article, and reading the article is free to anyone wishing to do so [4]. A journal may operate wholly under this model, or may use a hybrid model combining reader and author sponsorship [4].

With the growth of OA in the early 2000s, universities, research institutions and research funders started to adopt mandates and policies that require or request their researchers or grantees to make available their peer-reviewed research article output by depositing it in an OA repository. According to the Registry of Open Access Repository Mandates and Policies (ROARMAP), a searchable international registry charting the growth of institutional OA mandates and policies adopted worldwide, a total of 879 OA policies have formally been registered and adopted by entities as of Q3 2017 up from 127 registered and adopted policies at the registry’s creation in 2005 [5]. Most policies to date have been adopted by Europe (n = 543) followed by the Americas (n = 214), Asia (n = 58), Oceania (n = 40), and Africa (n = 24) [5]. To facilitate the identification of OA repositories for researchers who wish to self-archive, the initiative OpenDoar was launched in 2005. This initiative has now grown into a global directory of academic OA repositories [6]. It is worth noting that this registry is based on voluntary self-disclosure and since it is based in the UK, this might influence who knows about and uses the repository.

In 2012, the European Commission (EC) encouraged all European Union (EU) Member States to put publicly-funded research results in the public sphere to improve science and strengthen the knowledge-based economy [7]. In its recommendation of 17 July 2012 on access to and preservation of scientific information, the EC described the potentials benefits of OA as follows [8]:

- Acceleration of the research and discovery process, leading to increased returns on R&D investment
- Avoidance of the duplication of research efforts, leading to savings in R&D expenditure
- Enhanced opportunities for multi-disciplinary research, as well as inter-institutional and inter-sectorial collaborations
- Broader and faster opportunities for the adoption and commercialisation of research findings, generating increased returns on public investment in R&D and the potential for the emergence of new industries based on scientific information
- Increase in openness and transparency, thereby contributing to better policymaking and ultimately benefitting society and citizens.

With its implementation in 2014, the Horizon 2020 EU Framework Programme for Research and Innovation firmly anchored OA to scientific peer-reviewed publications in the Regulations and Rules of Participation [9]. The European & Developing Countries Clinical Trials Partnership (EDCTP), a public–public partnership between European and African countries and the European Union, supports collaborative research to accelerate the clinical development of new or improved interventions to prevent or treat poverty-related and neglected infectious diseases (PRDs) in sub-Saharan Africa. Being part of the H2020 framework programme, EDCTP has adopted the same OA policy.

In a preceding bibliometric assessment [10] of European and sub-Saharan African research output on PRDs, we found that the volume and citation impact of papers from Europe and sub-Saharan Africa has increased since 2003, as has collaborative research between Europe and sub-Saharan Africa. Research has demonstrated that collaborative papers are more highly cited than single-authored papers [11] and internationally collaborative papers are more highly cited than single-nation papers [12]. In this paper we wanted to assess the effect of publishing in OA and international collaboration within and between European and sub-Saharan African countries, specifically looking at citation impact and the likelihood of falling into the top 1% and top 10% most-cited papers in PRD research.

## Methods

### Data sources

The bibliometric indicators in this paper were produced using the Web of Science (WoS) database by Clarivate Analytics (formerly the IP&S business of Thomson Reuters), MEDLINE (Medical Literature Analysis and Retrieval System Online), and 1science oafindr. The WoS comprises bibliographic records of close to 52 million publications [13] published in scholarly journals between 1980 and 2017. These include peer-reviewed publications (i.e. papers, reviews, conference papers), but also other non-scientific documents (e.g. bibliographies, editorials, poetry, scripts). Peer-reviewed publications account for close to 31 million of these documents [13].

MEDLINE is a freely available life sciences and biomedical bibliographic database compiled by the United States National Library of Medicine. It can be accessed through the PubMed portal (https://www.ncbi.nlm.nih.gov/pubmed). In MEDLINE, subject-specific publications can be identified through the use of Medical Subject Headings (MeSH) terms. In a bibliometric context, these MeSH terms enable the computation of statistics on very precise fields by identifying highly relevant MeSH terms to delineate subject areas.

The 1science oafindr is a growing, comprehensive research discovery platform that currently comprises more than 27 million freely accessible OA publications and their metadata (e.g. digital object identifier, author, year of publication, and source title) [14]. All publications in oafindr, regardless of whether they are green or gold OA, are published in peer-reviewed journals. Gold and green OA publications can be identified using the URL where the publication is hosted [15].

### Construction of datasets

We first queried WoS for all relevant research publications on PRDs using an approach combining WoS and Medline for the period 1 January 2003 to 31 December 2015. We combined both databases because although Medline’s controlled vocabulary consisting of MeSH keyword is extremely powerful for retrieving documents relevant to specific topics, the database is not suited to performing full bibliometric analysis (i.e., not all institutional addresses are available; no easily usable citation links between documents exist for the computation of impact metrics). A list of search terms based on MeSH descriptors (S1 Table) was compiled to retrieve all relevant research publications on PRDs in Medline. A matching key between Medline and WoS was then used to retrieve all relevant documents covered in WoS. Although WoS covers a large share of Medline publications (>78% of publications published after 2000), some relevant publications covered only in WoS would have been missed using MeSH descriptors alone. Hence, an additional list of search terms (S2 Table) was developed to search for relevant documents in WoS that are not covered or captured by Medline. This list also includes a few highly specialised journals, the content of which was fully included under some PRDs. Finally, the content of both queries was combined to constitute the dataset for the analyses presented in this study. Only publications (including reviews) from peer-reviewed journals were included in the data extraction; hereafter referred to as ‘papers’. We excluded editorials, meeting abstracts, proceeding papers and other types of publications.

To assess whether the coverage achieved was adequately representative of the corresponding disease theme, precision was tested manually by examining random samples of papers from the dataset. The recall rate was estimated using the percentage of publications retrieved within benchmark specialist journals. Overall, tests demonstrated a precision level above 90% for each disease area, and a recall that ranged from 70% to 95%, depending on the disease-area dataset. It is worth noting that recall levels above 70% are usually quite difficult to achieve without compromising on precision.

To determine the OA status of publications included in the dataset, the 1science oafindr database was used. Using matching algorithms developed by 1science, each publication was tested against the more than 27 million peer-reviewed OA publications covered by 1science and were deemed as being freely available online when a match was found. oafindr covers a large share of all WoS publications freely available online (>80% as of March 2017) [14] and hundreds of thousands of additional publications are being added monthly, making it the most comprehensive database of OA publications suitable for large-scale bibliometric analyses.

We limited the OA analyses to the period 2011–2014 to avoid measuring citation metrics of older documents; those older documents may have received most of their citations before they were available in OA, a possibility that cannot be verified or controlled for with available data sources. For instance, a highly cited paper from 2005 may only recently have been made available in OA by its authors but most of the citations the publication received were before its release in OA. Furthermore, to enable a citation window of at least three years for every publication, only papers published in 2014 or earlier were considered in this study, to measure their impact properly. This analysis is referred to hereafter as “analysis on refined dataset”.

### Variables

The unit of analysis is PRD research papers published in peer-reviewed journals. Research impact is measured by three citation-based indicators: the average of relative citation scores, denoted by the average relative citation (ARC) and the top 1% and top 10% most cited papers, denoted as HCP1% and HCP10% respectively.

#### Dependent variables

**Relative Citation score (RC):** This score indicates the impact of a paper on the scientific community relative to expectations (i.e., the world level). The number of citations received by the paper is counted for the year in which it was published as well as for all subsequent years. To account for different citation patterns across subfields of science and over time, the paper’s citation count is divided by the average citation count of all papers that were published the same year in the same subfield. This results in the Relative Citation score (RC). The standardisation by year removes differences in the citation rates of papers due to variations in their citation window (i.e., the time they have had to accumulate citations) and changes in citations practices (i.e., increases/decreases in referencing practices, which could lead to decreases/increases in impact metrics). This indicator is based on full counting.

**Average of relative citations (ARC):** This score indicates the impact of a given entity (e.g., a country, an institution) on the scientific community relative to expectations (i.e., the world level). It is the average of the RCs of the papers belonging to the entity. An ARC value above 1 means that a given entity’s papers are cited more frequently than the world average, while a value below 1 means that its publications receive on average fewer citations than the world level.

**Highly cited papers (HCP10%):** The 10% most cited papers in the database are identified using the relative citation (RC) scores of papers, as presented above. This indicator is often used as a proxy to examine research excellence because of the high concentration of citations (close to 45%) in this elite group of papers [16].

**Highly cited papers (HCP1%):** Similar to the HCP10% indicator, the HCP1% instead focuses on the 1% most cited papers in the database. This indicator is even more focused on excellence, analysing the niche population of publications reaching this exclusive level.

#### Explanatory variables

**Open access:** This parameter is modeled as a binary value (1 or 0), indicating whether the publication is freely available online or not, as measured with the 1science oafindr database. OA status is also parameterized as a categorical variable, to study the effects on citation of gold, green and other OA. Some publications are served on multiple websites, or available in both gold OA and green OA, which could introduce noise into the analysis of citation impact by OA type. Accordingly, analyses referring to green and gold OA in this study are limited to green-only and gold-only publications; that is, these analyses consider publications that were retrieved from a green source only (i.e. publications available on platforms other than publishers’ websites) or a gold source only (i.e. publications available through publishers’ websites) but not both. The other OA category includes all OA that were not green-only and gold-only, so it includes papers that can be found in both green and gold OA, plus OA papers that were not yet assigned to gold and green OA as not all web domains have been coded to green and gold (about 80–90% of papers are coded to green or gold).

**International collaboration:** Following common practice this study adopts co-authorship involving authors from at least two different countries as an indicator of international research collaboration. Countries are assigned based on the institutional addresses linked to authors listed on the article. This parameter is modeled as a binary value (1 or 0), indicating whether or not the article is the fruit of an international collaboration. It was also considered whether to include this parameter as a scalar variable reflecting the number of countries involved, but previous work by the study team has concluded that binary modeling is more appropriate for this dimension [17].

International collaboration is also modeled as a categorical variable to assess the effect of the type of international collaboration on citation metrics. The following categories were used:

- **EUR-EUR co-publication status:** This parameter is a binary value (1 or 0), indicating whether or not the paper was a collaboration between at least two European (EUR) countries but without any collaborators from outside of Europe. European countries included in the analyses are listed in S3 Table. This variable was included in the model to assess whether North-North collaboration influences impact metrics in the context of PRD research
- **SSA-SSA co-publication status:** This parameter is a binary value (1 or 0), indicating whether or not the paper was a collaboration between at least two sub-Saharan (SSA) countries but without any collaborators from outside of sub-Saharan Africa. Sub-Saharan countries included in the analyses are listed in S3 Table. This variable was included in the model to assess whether South-South collaboration influences impact metrics in the context of PRD research.
- **EUR-SSA co-publication status:** This parameter is a binary value (1 or 0), indicating whether or not the paper was a collaboration between at least one European country and at least one country in sub-Saharan Africa but without any collaborators from outside sub-Saharan Africa and Europe. This variable was included in the model to assess whether North-South collaboration influences impact metrics in the context of PRD research.
- **EUR or SSA and other international co-publication status:** This parameter is a binary value (1 or 0), indicating whether or not the paper was a collaboration between at least one European country or at least one country in sub-Saharan Africa and any collaborators from outside Europe or sub-Saharan Africa. This variable was included in the model to assess whether European or sub-Saharan African collaboration with other regions influences impact metrics in the context of PRD research.

#### Control variables

**Number of authors:** This parameter models the total number of authors listed on a peer-reviewed paper, as a scaler. This parameter should be accounted for in the analysis given that more authors listed on a paper may have an impact on the number of citations obtained.

**Average of relative impact factors (ARIF):** The ARIF is a proxy indicator to measure the scientific quality or prestige of the journals in which an entity’s papers are published. Given its correlation with the ARC and that the journal impact factor has been well established to be a major factor affecting citations [18,19] the parameter was included in the model to control for competing explanations.

### Statistical analysis

Data were imported into Rstudio running R (version 3.3.1) and Stata version 14.2 software (StataCorp, College Station, TX, USA) from Microsoft Excel files. For the descriptive analyses, differences between international collaboration, OA and disease area were tested using two-sided Pearson Chi-square test for categorical outcomes, using α = 0.05 as the significance level. Generalised linear modelling (GLM) was used to quantify the relationship between the dependent variables ARC, HCP1% and HCP10% and the independent variables OA and international collaboration adjusting for number of authors and the average relative journal impact factor (ARIF), two potential confounders associated with the dependent and independent variables of interest in this study. Regression models show how much variance of the dependent variables can be explained by the independent variable of interest holding all other variables constant. For the continuous outcome variable ARC, GLM was modeled using the gamma distribution and the log link in light of the right skewedness of the outcome variable. Log-transformed ordinary least-squares (OLS) regression was not considered to be appropriate given the observed heteroskedasticity in the data. The HCP1% and HCP10% were modeled using the binomial distribution and the logit link with the reported fixed effects coefficient as odds ratios and 95% confidence intervals (95% CI). In the gamma and binomial regressions that assessed the effect of OA and international collaboration on the citation metrics (so called “model 1”), the independent variable ARIF was log 10 transformed as this provided the best model fit. In the OA and international type- specific gamma and binomial regression models (so called “model 2”), the ARIF was modelled both as log 10-transformed and as a categorical variable. Given the very similar model fit and coefficients that were obtained, for ease of interpretation, we chose to include ARIF as a categorical variable. For the categorisation of the ARIF variable in model 2, the ARIF distribution was divided into five bins of equal size and the median ARIF per bin was calculated. Furthermore, for ease of interpretation in model 2 we created five categories for the independent variable “number of authors” variable based on the distribution of number of papers per category of number of authors.

## Results

**Fig 1:**
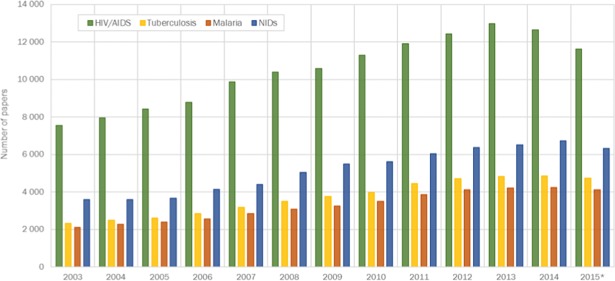
World trends in research output in poverty related diseases, 2003–2015. (*Because MeSH Subject Headings were used to define the subject areas and these are not fully provided for MEDLINE publications published in the most recent years, data are incomplete for 2015).

The final combined WoS-Medline dataset contained 277,744 peer-reviewed OA and non- OA papers on PRDs published between 2003 and 2015. The output of research papers on PRDs has been steadily increasing across all disease areas since 2003, averaging a 6.1% increase per year (Fig 1). This is higher than the average annual growth in output of papers in medical sciences at world level (4.2%) between 2003 and 2015.

**Fig 2:**
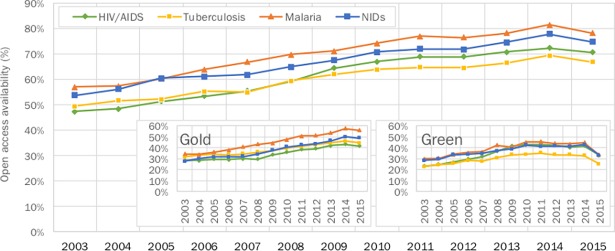
Open access availability of papers, 2003–2015.

Fig 2. shows that papers across all PRD areas are increasingly becoming available in OA, with a more pronounced upward trend in gold OA than green OA. In 2003, less than half of research papers across all disease areas were available in OA, except for malaria at 52.3%. By 2015, more than 75% of the research papers on malaria and neglected infectious diseases (NIDs) were accessible in OA, while HIV/AIDs sat at 71% and tuberculosis at 67%.

**Fig 3:**
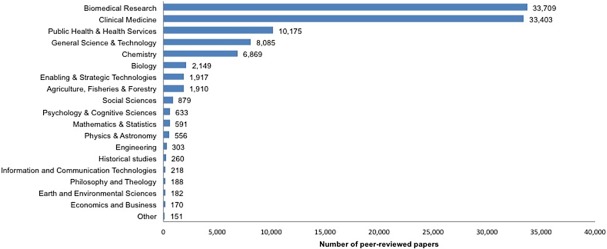
Number of peer-reviewed papers on PRDs in dataset, by field of science 2011–2014.

To assess the effect of publishing in OA and of international collaboration on citation impact and likelihood of falling into the top 1% and top 10% most cited papers in PRD research, we refined our dataset to peer-reviewed OA and non-OA papers on PRDs published between 2011 and 2014. As shown in Fig 3. a total of 102,348 peer-reviewed papers were published during this period and these papers were published in journals across a variety of fields of science, but primarily concentrated in biomedical research (32.9%) and clinical medicine (32.6%). The sub-Saharan African research output share (i.e., only including SSA authors) in the refined dataset was 14.9% (n = 15,230 papers). Of note, only fields of science with more than 300 publications in the dataset are shown—see Science-Metrix classification of field of science used [http://www.science-metrix.com/en/classification].

**Fig 4:**
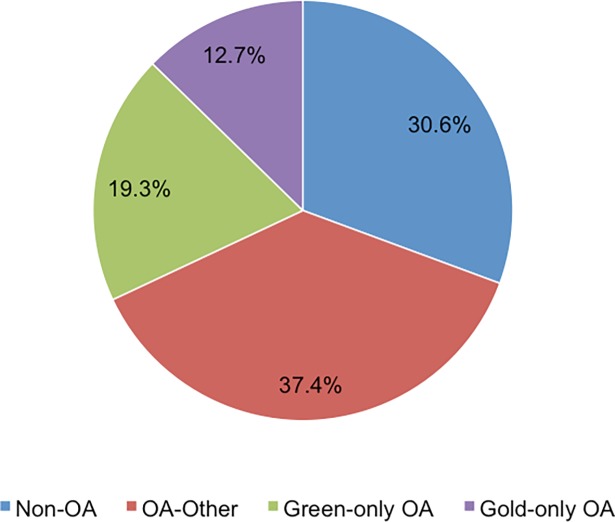
Poverty-related disease publications available in open access, 2011–2014.

In total, 70,993 (69.4%) papers in the refined dataset were available in OA, including 12.7% (n = 12,999) in gold-only OA; 19.3% (n = 19,725 papers) in green-only OA; and 38.3% of OA papers (n = 38,269) in both green and gold or via other OA (i.e. through aggregator sites such as PubMed Central) (Fig 4). The other OA category includes all OA that were not green-only and gold-only, so includes papers that can be found in both green and gold OA, plus OA papers that were not yet assigned to gold and green OA. Most sub-Saharan African research output was available in OA (78.5%), including 11.0% (n = 1,675 papers) in gold-only; 16.1% (n = 2,453 papers) in green-only; and 51.4% (n = 7,824 papers) available in other OA.

**Fig 5:**
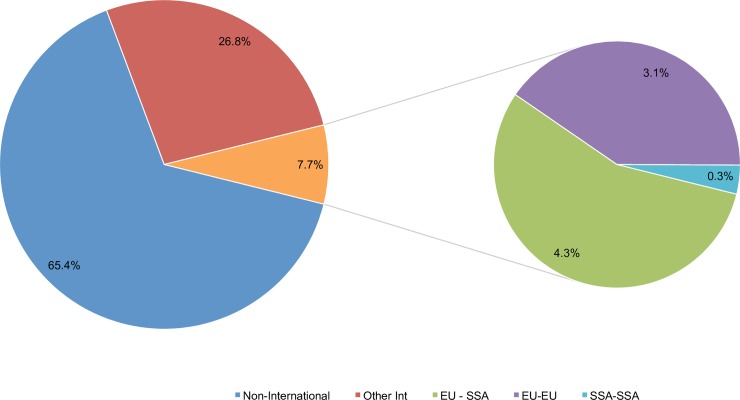
International collaboration in poverty-related disease publications, 2011–2014.

In total, 35,383 (34.6%) of the papers in the refined dataset were published in international collaboration (Fig 5). Almost eight percent (n = 7,921) of papers published in international collaboration fell into the three regional sub-categories examined, indicating that most internationally collaborative research (n = 27,462 papers; 26.8%) involves other countries excluded by these category variables (e.g. the United States). Of the three collaboration sub-types assessed, European collaboration with sub-Saharan African institutions was the most common (n = 4,413 papers; 4.3%), followed by European-European collaboration (n = 3,208 papers, 3.1%) (Fig 5). Collaborations between institutions in two or more sub-Saharan African countries were much rarer, having led to only 300 (0.29%) publications between 2011 and 2014. Among the papers involving no international collaboration (n = 66,965 papers) almost 5% (n = 3,244 papers) originated from sub-Saharan Africa with most of these deriving from Southern Africa (n = 1,728 papers) (S3 Table).

Research in HIV/AIDS made up the largest proportion of the refined dataset at 49,128 papers (Table 1), followed by NIDs (n = 25,168), tuberculosis (n = 18,541) and malaria (n = 16,238). The degree of international collaboration and the profile of collaboration (by proportion of publication output) varied significantly across disease types (p<0.001). International collaboration was highest in malaria research (46.1%) and lowest in tuberculosis research (31.8%). Malaria research also had the highest proportion of collaborative papers across the three-different co-publication sub-groups: EUR-SSA (8.7%), EUR-EUR (3.6%), and SSA-SSA (0.52%) (Table 1).

**Table 1:** Distribution of papers by disease and collaboration type, 2011–2014.

Disease	Papers (n)*	International collaboration (n, %)		EUR-SSA (n, %)		EUR-EUR (n,%)		SSA-SSA (n, %)	
HIV/AIDS	49,128	16,472	(33.5%)	1,978	(4.0%)	1,407	(2.9%)	153	(0.31%)
Malaria	16,238	7,492	(46.1%)	1,420	(8.7%)	589	(3.6%)	85	(0.52%)
NIDS	25,168	8,651	(34.4%)	883	(3.5%)	777	(3.1%)	53	(0.21%)
Tuberculosis	18,541	5,889	(31.8%)	798	(4.3%)	631	(3.4%)	45	(0.24%)
Total	109,075	38,504	(35.3%)	5,079	(4.7%)	3,404	(3.1%)	336	(0.31%)

* One paper can cover more than one disease area and thereby can be included in more than one disease specific analysis

Limiting our analysis to research output including at least one author from sub-Saharan Africa, and to the 2011–2014 period (n = 17,359; 15.9%) showed that most research output in sub-Saharan Africa is produced with an international partner (n = 13,697 papers; 78.9%). This included 5,079 papers (37.1%) co-authored with a European partner (excluding any additional collaboration with countries outside Europe and sub-Saharan Africa), and 366 papers (2.5%) that included another sub-Saharan African partner (excluding any additional collaboration with countries outside sub-Saharan Africa).

Over the 2011–2014 period, nearly 70% of papers on PRDs were published in OA (Table 2). The degree of OA varied significantly across disease types (p<0.001). Papers on malaria were most commonly published in OA (75.9%). When assessing the type of OA publishing, green-only OA was more common than gold-only OA across all diseases; however, the gap between green and gold varied. Green OA publications were most common in HIV/AIDS (21.5%), which is also the disease group with the lowest proportion of gold OA publications (11.1%). By contrast, tuberculosis research was the least commonly published in green OA (15.6%) but the most commonly published in gold OA (14.6%).

**Table 2:** Distribution of papers by disease and open access, 2011–2014.

Disease	Papers (n)*	Open access (n,%)		Green OA (n,%)		Gold OA (n,%)	
HIV/AIDS	49,128	32,811	(66.8%)	10,543	(21.5%)	5,459	(11.1%)
Malaria	16,238	12,321	(75.9%)	2,710	(16.7%)	2,044	(12.6%)
NIDS	25,168	18,070	(71.8%)	4,678	(18.6%)	3,610	(14.3%)
Tuberculosis	18,541	11,825	(63.8%)	2,945	(15.9%)	2,709	(14.6%)
Total	109,075	75,027	(68.8%)	20,876	((19.1%)	13,822	(12.7%)

* One paper can include more than one disease area and thereby can be included in more than one disease specific analysis

OA papers on PRD research showed significant citation advantage over non-OA PRD papers. For the 2011–2014 period, the overall ARC of OA papers was 42% greater than the ARC of non-OA papers (Exp(b) 1.42, 95% CI: 1.38–1.46) (Table 3, model 1). This significant effect was also observed in the multivariable analyses when adjusted for international collaboration, number of authors on a paper and ARIF. Looking into the types of OA, papers published in green-only OA (Table 3, model 1) were 29% more cited (Exp(b) 1.29, 95% CI 1.26–1.32); those in gold-only OA 6% (Exp (b) 1.06, 95% CI 1.03–1.08), and those in other OA 21% (exp(b) 1.21, 95%CI 1.18–1.23) compared to papers published in non-OA. Likewise, in the univariable regression analysis, PRD papers published in international collaboration were 50% more cited (Exp(b) 1.50, 95% CI 1.47–1.54) compared to those not published in international collaboration. Although the citation advantage was reduced in the multivariable regression analysis when controlling for publishing in OA, number of authors and ARIF, papers published in international collaboration still received 13% more citations compared to papers that were not published in an international collaboration (Table 3, model 1). Papers published as part of a European-sub-Saharan African or European-European collaboration received 10% and 18% more citations, respectively, compared to papers that were not published in an international collaboration (Table 3, model 2). Papers published within a sub-Saharan African collaboration received 26% (Exp(b) 0.74, 95% CI 0.64–0.85) fewer citations.

**Table 3:** Univariable and multivariable generalised linear model derived exponentiated coefficients (Exp (b)) and 95% confidence intervals (CIs) to assess independent effects of publishing in open access (OA) and international collaboration on the average relative citation (ARC) score.

	Univariable analyses		Multivariable analyses	
Variable	Crude Exp (b)	95% CI	Adjusted Exp (b)	95% CI
***Model 1***				
Open Access (no, yes)	1.42	1.38–1.46	1.24	1.22–1.26
International collaboration (yes no)	1.50	1.47–1.53	1.13	1.11–1.15
Number of authors	1.06	1.05–1.06	1.03	1.02–1.03
Average relative impact factor (ARIF) log10 transformed	7.24	6.94–7.56	6.34	6.19–6.48
Model 2				
*Open Access categories*				
No OA	Ref		Ref	
Gold-only OA	1.10	1.06–1.14	1.06	1.03–1.08
Green-only only	1.51	1.46–1.56	1.29	1.26–1.32
Other OA	1.49	1.45–1.53	1.21	1.18–1.23
*International collaboration categories*				
No co-publication	Ref		Ref	
EUR-SSA co-publication	1.33	1.27–1.40	1.10	1.06–1.14
EUR-EUR co-publication	1.47	1.38–1.55	1.18	1.13–1.24
SSA-SSA co-publication	0.69	0.57–0.83	0.74	0.65–0.86
EU or SSA and other international co-publication	1.54	1.51–1.58	1.17	1.15–1.19
*Number of co-authors*				
0–4	Ref		Ref	
5–8	1.16	1.14–1.18	1.08	1.06–1.10
9–12	1.48	1.44–1.52	1.23	1.20–1.26
13–20	2.17	2.08–2.25	1.56	1.50–1.61
>20	5.22	4.78–5.69	3.04	2.81–3.28
*Average relative impact factor*				
Low (median point ARIF 0.38)	0.34	0.33–0.35	0.38	0.37–0.39
Medium (median point ARIF 0.68)	0.62	0.61–0.64	0.63	0.62–0.65
Average (median point ARIF 0.88)	Ref		Ref	
Medium (median point ARIF 1.16)	1.12	1.09–1.15	1.11	1.09–1.14
High (median point ARIF 1.67)	1.96	1.91–2.01	1.79	1.75–1.83

As shown in model 1 and model 2, increases in the number of authors and the ARIF were significantly associated with an increase in the adjusted predictions of the ARC. Hence papers with more authors and papers published in high impact journals receive higher numbers of citations.

As shown in Table 4 multivariable analyses: model 1, the odds of a PRD article falling in the top 1% and top 10% most cited in its field of science was significantly higher for papers published in OA (83% and 56% respectively) and international collaboration (28% and 23% respectively), after controlling for the confounding effects of number of authors and ARIF. We next examined in the multivariable regression analysis the effect of publishing in green-only OA only and gold-only OA, while for international collaboration, we examine more specifically international collaborations 1) between European and sub-Saharan African countries; 2) amongst European countries, and 3) amongst sub-Saharan African countries. The results of the multivariable analyses showed that publishing in gold-only OA was only associated with significantly higher odds for HCP10% (OR 1.30, 95% CI 1.20–1.40) but not for HCP1% (OR 1.14, 95% CI: 0.88–1.47) (Table 4). However, publishing in green-only OA was significantly associated with higher odds for falling in both HCP1% (OR 1.51, 95% CI 1.24–1.85) and HCP10% (OR 1.63, 95% CI 1.43–1.63).

**Table 4:** Univariable and multivariable generalised linear model derived odds ratios (OR) and 95% confidence intervals (CIs) to assess independent effects of publishing in open access (OA) and international collaboration to fall into the top 1% or top10% most cited peer-reviewed papers on PRDs.

	HCP 1%				HCP10%			
	Univariable analyses		Multivariable analyses		Univariable analyses		Multivariable analyses	
Variable	Crude OR	95% CI	Adjusted OR	95% CI	Crude OR	95% CI	Adjusted OR	95% CI
***Model 1***								
Open Access (no, yes)	2.25	1.91–2.65	1.83	1.53–2.18	1.98	1.88–2.08	1.56	1.48–1.64
International collaboration (no, yes)	2.33	2.06–2.63	1.28	1.11–1.48	1.97	1.89–2.04	1.23	1.18–1.29
Number of authors	1.09	1.08–1.11	1.05	1.04–1.06	1.10	1.09–1.11	1.07	1.06–1.07
Average relative impact factor (ARIF) log10 transformed	62.11	52.54–73.42	46.45	38.65–55.82	74.67	67.74–82.30	63.04	57.16-69-53
***Model 2***								
*Open Access categories*								
No OA	Ref		Ref		Ref		Ref	
Gold-only OA	1.31	1.02–1.69	1.14	0.88–1.47	1.35	1.26–1.46	1.30	1.20–1.40
Green-only OA	2.12	1.74–2.58	1.51	1.24–1.85	2.05	1.93–2.18	1.53	1.43–1.63
Other OA	2.63	2.22–3.13	1.69	1.42–2.02	2.16	2.05–2.28	1.57	1.49–1.67
*International collaboration categories*								
No co-publication	Ref		Ref		Ref		Ref	
EUR-SSA co-publication	1.61	1.20–2.16	1.00	0.75–1.36	1.67	1.52–1.83	1.13	1.02–1.24
EUR-EUR co-publication	2.05	1.51–2.78	1.17	0.86–1.60	2.11	1.92–2.33	1.38	1.24–1.53
SSA-SSA co-publication	0.47	0.07–3.36	0.56	0.08–4.00	0.37	0.20–0.70	0.40	0.21–0.75
EU or SSA and other international co-publication	2.50	2.20–2.84	1.30	1.13–1.50	2.02	1.93–2.10	1.28	1.22–1.34
*Number of co-authors*								
1–4	Ref		Ref		Ref		Ref	
5–8	1.08	0.91–1.28	0.96	0.81–1.15	1.24	1.18–1.31	1.11	1.06–1.18
9–12	1.92	1.60–2.31	1.40	1.15–1.70	1.89	1.79–2.01	1.44	1.35–1.53
13–20	4.42	3.63–5.39	2.48	1.42–3.07	3.80	3.54–4.07	2.46	2.28–2.66
>20	22.22	17.11–27.79	9.35	7.31–11.96	11.07	9.74–12.58	6.21	5.39–7.15
*Average relative impact factor*								
Low (median point ARIF 0.38)	0.05	0.02–0.11	0.07	0.03–0.15	0.11	0.09–0.12	0.13	0.12–0.15
Medium (median point ARIF 0.68)	0.30	0.21–0.43	0.30	0.21–0.42	0.31	0.28–0.34	0.30	0.28–0.33
Average (median point ARIF 0.88)	Ref		Ref		Ref		Ref	
Medium (median point ARIF 1.16)	1.21	0.96–1.52	1.23	0.98–1.44	1.24	1.17–1.32	1.25	1.18–1.33
High (median point ARIF 1.67)	5.44	4.51–6.55	4.62	3.83–5.58	3.45	3.27–3.65	3.22	3.04–3.40

Papers published in collaboration between European and sub-Saharan African countries (OR 1.13, 95% CI 1.02–1.24) or amongst two or more European countries (OR 1.38, 95% CI 1.24–153) were significantly more likely to be in the top 10% most cited papers compared to papers that were not published in international collaboration. This advantage was not observed for HCP1%. In contrast, papers published with collaborators from two or more countries in sub-Saharan Africa had 60% lower odds of falling in HCP10% (OR 0.40, 95% CI 0.21–0.75) and 44% lower odds of falling in HCP1% (OR 0.56, 0.08–4.00), although the latter reduction was not statistically significant.

Similar to the observed citation advantage, having more authors listed on a paper and publishing in high impact journals was associated with a significant increase in the odds ratio for HCP1% and HCP10% in the uni- and multivariable analyses.

## Discussion

With the creation of the first OA journals in 2000 the growth rate of OA across many different disciplines has steadily increased [20]. In 2013, the total percentage of OA papers available was estimated at 24% of English-language scholarly documents accessible on the Web, with significant differences between scholarly fields [21]. For example, the percentage of OA papers in the field of Medicine was estimated at 26% (95% CI ± 8.5) and at 43% (95% CI ± 8.7) for multidisciplinary fields.

Our results show that in the field of PRDs, most researchers have already made the switch from paywalled to OA publishing with more than two-thirds of the scholarly papers on PRDs now available in OA. This observation is consistent with the growth of OA mandates and policies adopted worldwide since 2005 by many universities, research institutions and research funders [5] that require or request researchers to publish their results in OA.

Our results further show that since 2003 OA accessibility in the malaria research field has been higher compared to other PRDs. Malaria received special attention in the 2000 Millennium Development Goals due to its high burden of disease in the (sub)-tropics and associated economic costs [22]. As a result, at the start of the 21st century many philanthropic organisations, governments and international organisations started to fund research in malaria-endemic countries to develop effective intervention tools to support the elimination efforts [23].

However, as pointed out by B. Knols “an unfortunate consequence of the traditional publishing system has been that in order to seek maximum global visibility and publicity for their work, leading researchers working in low to middle-income countries (LMICs) have until recently had no choice but to publish in developed country paywalled journals, even though this has meant that readers in their own country would not have easy access to published results” [24]. The move to the OA publishing model has improved matters considerably. As reflected in our results, many malaria researchers in LMICs quickly embraced the first OA journals in the malaria field. This was probably facilitated by the fact that BioMed Central, which includes OA journals such as the *Malaria Journal* (launched in 2002) and *Parasite and Vectors* (launched in 2008) made a special effort to attract papers from authors from LMICs [23].

Furthermore, with the increase in OA, many sub-Saharan African countries have acknowledged the need for their research communities to have access to research and have consequently have launched repositories to increase access through self-archiving of research papers [20]. In 2014, over 100 institutions participated in the “Open access: Knowledge sharing and sustainable scholarly communication in Kenya, Tanzania and Uganda” project, which resulted in 31 fully operational OA repositories in Kenya, Tanzania and Uganda, 29 repositories under construction, one OA journal launched and 13 more OA journals in the process of being set up [25]. Such developments are important steps toward leveling the research playing field between European and sub-Saharan African countries [20].

With the steady increase in OA over the past two decades, the debate on whether or not publishing in OA has a greater research impact has become a hot topic in the scientific community and one intensively debated [2,4,21,26,27,28]. In a recent review article on the academic, economic and societal impacts of OA, Tennant et al [20] indicated that there is a “general tendency identified by studies to date that there is at least some association between OA publishing and increased citation counts across most disciplines”. Across the 70 studies included in his review 66% found a citation advantage; 10% were inconclusive or found a non-significant advantage, and 24% found no citation advantage [20]. The citation advantages in these studies ranged from +36% to +600% [20]. As pointed out by Tennant et al not all studies adjusted for relevant predictors but when taking these into account the citation advantage would become much smaller (+8%) [20]. Similarly, Piwowar et al recently reported an unadjusted OA citation advantage of 18% [28].

Our results are closely aligned with these observations. The crude regression results showed that publishing in OA carries a significant citation advantage of 42% over non-OA. Adjusting the regression for international collaboration, number of authors and ARIF did weaken the association between OA and ARC slightly but a significant citation advantage of 24% (95% CI 1.22–1.26) could still be observed. In addition to the citation advantages, we also observed that the adjusted odds of falling in the top 1% and top 10% most cited papers were 1.83 (95%CI 1.53–2.18) and 1.56 (95%CI 1.48–1.64) times higher, respectively, for papers published in OA.

In our analysis, green OA carried a significantly higher citation advantage (29% - 95%CI 1.26–1.32) compared to gold OA (6% - 95% CI 1.03–1.08) as well as significantly higher odds of falling in the top 10% most cited papers. Piwowar et al also reported this difference in citation advantage between green OA and gold OA. They showed an unadjusted citation advantage of 33% (ARC 1.33) for green OA while gold OA was cited 17% below world average (ARC 0.83) [28].

A possible explanation for the difference in observed citation advantage between green and gold OA could be that gold OA journals are among the relatively newer and less established journals. The difference in citation advantage would therefore not be a phenomenon related primarily to OA, but to the age and establishment of the journal. Another hypothesis to potentially explain the citation gap is that many gold OA journals charge authors a so-called article-processing fee (APC) for publication, which could put a financial constraint on the author. A constraint on providing green OA is whether or not the author actually chooses to self-archive [29] and adhering to publishers’ OA policies (i.e., embargo periods). Interestingly, Piwowar et al reported that gold OA was cited 17% below world average [28] while we observed that gold OA was cited 6% above world average. Possible explanations for this divergence could be the result of differences in statistical analyses (unadjusted versus adjusted models) or due to a more restrictive definition of gold OA that Piwowar applied (i.e., only included articles in OA journals that are indexed by the Directory of Open Access Journals) [28].

In conjunction with publishing in OA, collaboration has also increasingly become the norm and, as pointed out by Wagner et al [30], “global collaboration continues to grow as a share of all scientific cooperation, measured as co-authorship of peer-reviewed, published papers”. Between 1990–2011 the percentage of research papers that are internationally co-authored more than doubled from 10.1% to 24.6%. In our analyses on PRD research we observed an international collaboration rate of nearly 35% over the 2011–2014 period. Of note is that sub-Saharan African research output on PRDs is mostly the result of international collaboration (79%) with very limited output (2.5%) coming from collaboration amongst sub-Saharan African partners alone. As point out by Breugelmans et al [10], this is in part due to a lack of critical mass of researchers in sub-Saharan Africa with limited South-South collaboration. Even where local scientific leadership exists, this is mainly established through external research funding that influences the constitution (i.e., nature) of collaborating partners.

Collaborative research has shown to lead in general to higher citation impact in many scientific fields [11,31] but this advantage seems to be dependent on the type of research collaboration [32] such as international versus inter-institutional collaboration [18]. Our multivariable regression analysis showed that PRD papers published in international collaboration were 13% more cited (Exp(b) 1.50, 95% CI 1.47–1.54) and were more likely to fall in the top 1% or top 10% of most cited papers compared to papers not published in international collaboration. Papers published as part of a European-wide or European-sub-Saharan African collaboration received 18% and 10% more citations, respectively, and were significantly more likely to be in the top 10% most cited papers compared to papers that were not published in international collaboration. The advantage of these type-specific international collaborations was not observed for the top 1%. This is most likely due to the low number of papers falling in the top 1% most cited papers for each international collaboration type (i.e., EUR-EUR, 50 papers; EUR-SSA, 46 papers; and SSA-SSA, 1 paper).

Of note is that papers published with collaborators from two or more countries in sub-Saharan Africa but no partner from a high-income country were less likely (26%) to be cited or to fall in the top 1% (OR 0.59 95%CI 0.08–4.00) or top 10% (OR 0.40 95%CI 0.21–0.75) of most cited papers. These findings indicate that collaborative research restricted to institutions in sub-Saharan Africa seems to be less impactful than research produced in other types of collaborations. While this conclusion is of a purely statistical nature, it merits further exploration to better understand the underlying patterns causing this phenomenon. We show that international scientific collaboration with a European partner is particularly advantageous for countries in sub-Saharan Africa.

Since 2003, several European countries have increased their research interest in PRDs with increasing collaborations amongst themselves and with sub-Saharan African countries [10]. The impact of this research has now started to emerge and might be attributed partly to research funders such as EDCTP, European Union Research Framework Programmes, Medical Research Council (MRC)-UK, Department for International Development (DFID)-UK, and the Swedish International Development Cooperation Agency, amongst others, that actively promote research between European and sub-Saharan African countries. While North-South collaborations have been actively supported to increase the critical mass of researchers in sub-Saharan Africa, the external research funding often influences the constitution of collaborating partners. Hence, an unfortunate consequence of these funding policies could have been that less focus has been put on the development of pure South-South collaborations. If South-South collaborations are to yield impactful research, it is important to further understand how exactly international collaboration with European partners and others exerts an advantage on research impact (e.g. through improved access to funding, specialised expertise, administrative support, broadened pool of researchers to get cited by due to increased visibility of the research, etc.) as well as the role of the individual researcher and local research institute so that appropriate funding and support policies can be developed.

To the best of our knowledge, this study is the largest scale measurement of OA availability performed in the PRD research areas to date. A strength of the analysis is that it controls for confounding factors such as the number of authors and the quality of the journal in which research is published, providing a more robust understanding of the independent effects of publishing in OA and in international collaboration. However, the study is limited in its ability to shed light on the trends in PRD research worldwide, given its focus on European and sub-Saharan African countries. The finding that 26.8% of collaboration in PRD research occurred between authors outside of the population studied suggests that there are further collaboration dynamics with other regions to be explored. Expanding the geographical scope of such analyses to include countries such as the United States (a major funder and contributor to PRD research) would provide a more complete picture of the landscape dynamics; Sud et al [32] have already pointed out that international collaboration with the United States seems to increase impact. Future research should focus on the presence of specific European and sub-Saharan African countries in combination with the US to disentangle the underlying patterns of international collaboration on research impact.

Another limitation of the study is that since the focus of this study is on PRD research the results are valid for this research area, but it is unclear whether the results can be generalised to other areas.

A third limitation of this study is that the multivariable analysis did not control for sources of funding. Funding acknowledgments are available in WoS since 2009. However, to date the shares of papers with a funding acknowledgment section indexed in WoS is only 72%. Furthermore, and most importantly, funding acknowledgments do not specify the level of the funding which would probably drive any effect linked to funding sources. Therefore, while controlling for the sources of funding would be pertinent, there is currently no satisfactory way to account for this.

Lastly, the mechanisms through which green OA exerts a stronger effect on research impact metrics than gold OA are not clear and merit further investigation in order to derive policy or funding-relevant recommendations.

## Conclusions

Our results lend support to the existence of a real, measurable citation advantage for OA and international collaboration, with lower confidence interval bounds of 11% and 22%, respectively. The benefits of OA as demonstrated in this study bolster the arguments to fight for OA, as it may ultimately support economic growth and development by increasing access to research by actors in resource constrained settings. It also demonstrates that international collaboration is in some cases associated with higher impact; however, further research is required to understand the mechanisms behind why collaborations restricted to sub-Saharan African institutions showed lower research impact relative to research conducted between European institutions, or between European and sub-Saharan African institutions.

It would also be interesting to measure whether collaborations restricted to sub-Saharan African countries produce higher-impact publications compared to research conducted by one sub-Saharan African country. Questions that should be addressed is whether the observed lower impact of research publications restricted to sub-Saharan African countries is a reflection of the quality of the research outputs and to which extent limited local investment in research affect the research scope and capacity of researchers conducting locally funded research. It may also be argued that a large percentage of research publications restricted to sub-Saharan African countries may also be subsets of multicenter international studies published by African partners in low impact factor journals including some less established African medical journals.

Having access to OA starts to level the playing field between European and sub-Saharan African scientists but if research institutes in the Global South aspire to match their counterparts in the North, more political commitment and increased capacity-building is needed across the board to enable existing and new research communities in sub-Saharan Africa to not only sustain the current research output but to specifically promote increased research output from South-South collaborations on diseases and needs relevant to the specific regions. This underpins the need to bolster funding for health research and development by African governments as major contributors and to improve policymakers’ understanding of the value of research to drive national health priority-setting [10,33].

## Supporting information

S1 Table MeSH descriptors selected to define diseases. (DOCX)

S2 Table Full-text searches in WoS abstracts, author keywords and titles for selected diseases. (DOCX)

S3 Table European and sub-Saharan African countries included in the bibliometric analyses. (DOCX)
