# Supplementary material for: Scientific impact increases when researchers publish in open access and international collaboration: A bibliometric analysis on poverty-related disease papers
Source: PLoS One. 2018 Sep 19;13(9):e0203156. doi: 10.1371/journal.pone.0203156 (PMC6145557; doi:10.1371/journal.pone.0203156)
Supplement: S2 Table — (DOCX) [file pone.0203156.s002.docx]

**S2 Table. Full-text searches in WoS abstracts, author keywords and titles for selected diseases**
