# Supplementary material for: Scientific impact increases when researchers publish in open access and international collaboration: A bibliometric analysis on poverty-related disease papers
Source: PLoS One. 2018 Sep 19;13(9):e0203156. doi: 10.1371/journal.pone.0203156 (PMC6145557; doi:10.1371/journal.pone.0203156)
Supplement: S3 Table — (DOCX) [file pone.0203156.s003.docx]

**S3 Table. European and sub-Saharan African countries included in the bibliometric analyses**

| ***European Countries*** | |
| --- | --- |
| Austria | Latvia |
| Belgium | Lithuania |
| Bulgaria | Luxembourg |
| Croatia | Malta |
| Cyprus | Netherlands |
| Czech Republic | Norway |
| Denmark | Poland |
| Estonia | Portugal |
| Finland | Romania |
| France | Slovakia |
| Germany | Slovenia |
| Greece | Spain |
| Hungary | Sweden |
| Ireland | Switzerland |
| Italy | United Kingdom |
| ***Central Africa*** | ***East Africa*** |
| Angola^[[1]](#footnote-1)^ | Burundi |
| Cameroon | Djibouti |
| Central African Republic | Eritrea |
| Chad | Ethiopia |
| Congo | Kenya |
| Democratic Republic of the Congo | Rwanda |
| Equatorial Guinea | Somalia |
| Gabon | South Sudan |
| São Tomé and Príncipe | Sudan |
| Cameroon | Tanzania |
| Central African Republic | Uganda |
| ***Southern Africa*** | ***West Africa*** |
| Botswana | Benin |
| Comoros | Burkina Faso |
| Lesotho | Cape Verde |
| Madagascar | Côte d'Ivoire |
| Malawi | Ghana |
| Mauritius | Guinea |
| Mozambique | Guinea Bissau |
| Namibia | Liberia |
| Seychelles | Mali |
| South Afric | Mauritania |
| Swaziland | Niger |
| Zambia8 | Nigeria |
| Zimbabwe | Senegal8 |
|  | Sierra Leone |
|  | The Gambia8 |
|  | Togo |

1. For the bibliometric analyses, Angola was included in the Central Africa region according to a World Bank 2012 regional classification. It is recognised that Angola is member of the Southern African Development Community. [↑](#footnote-ref-1)
